# Functional divergence of conserved developmental plasticity genes between two distantly related nematodes

**DOI:** 10.1038/s41598-025-14207-5

**Published:** 2025-08-05

**Authors:** Sara Wighard, Hanh Witte, Ralf J. Sommer

**Affiliations:** 1 Department for Integrative Evolutionary Biology, Max Planck Institute for Biology, Max-Planck-Ring 9, 72076 Tübingen, Germany; 2 Institute of Molecular Biotechnology of the Austrian Academy of Sciences (IMBA), Vienna BioCenter (VBC), 1030 Vienna, Austria

**Keywords:** Polyphenisms, Developmental plasticity, *Allodiplogaster sudhausi*, *Pristionchus pacificus*, *eud*-*1*/sulfatase, Nuclear-hormone-receptors, Gene divergence, Evolution, Zoology

## Abstract

Genes diverge in form and function in multiple ways over time; they can be conserved, acquire new roles, or eventually be lost. However, the way genes diverge at the functional level is little understood, particularly in plastic systems. We investigated this process using two distantly related nematode species, *Allodiplogaster sudhausi* and *Pristionchus pacificus*. Both these nematodes display environmentally-influenced developmental plasticity of mouth-form feeding structures. This phenotype can be manipulated by growth on particular diets, making them ideal traits to investigate functional divergence of developmental plasticity genes between organisms. Using CRISPR-engineered mutations in *A. sudhausi* mouth-form genes, we demonstrate examples of the various ways ancestral genes regulate developmental plasticity and how these roles can progressively diverge. We examined four ancestral genes, revealing distinct differences in their conservation and divergence in regulating mouth phenotype in both species. Loss of certain genes results in similar developmental disruptions in both species, while for others they are distinct. Additionally, two ancestral genes retain their functions as switch genes, which completely prevent a phenotype, and the other two display quantitative effects, with knock-outs in these genes displaying intermediate phenotypes. Remarkably, despite the evolutionary distance, all genes examined were involved in mouth-form regulation. Finally, key sulfatase-encoding genes act downstream of the other genes, suggesting they play a major role in mouth-form plasticity. Together, this study represents the first mutant-based functional analysis of the evolution of developmental plasticity between two highly diverged species, offering new insights into the genetic mechanisms underlying phenotypic evolution.

## Introduction

**Fig. 1:**
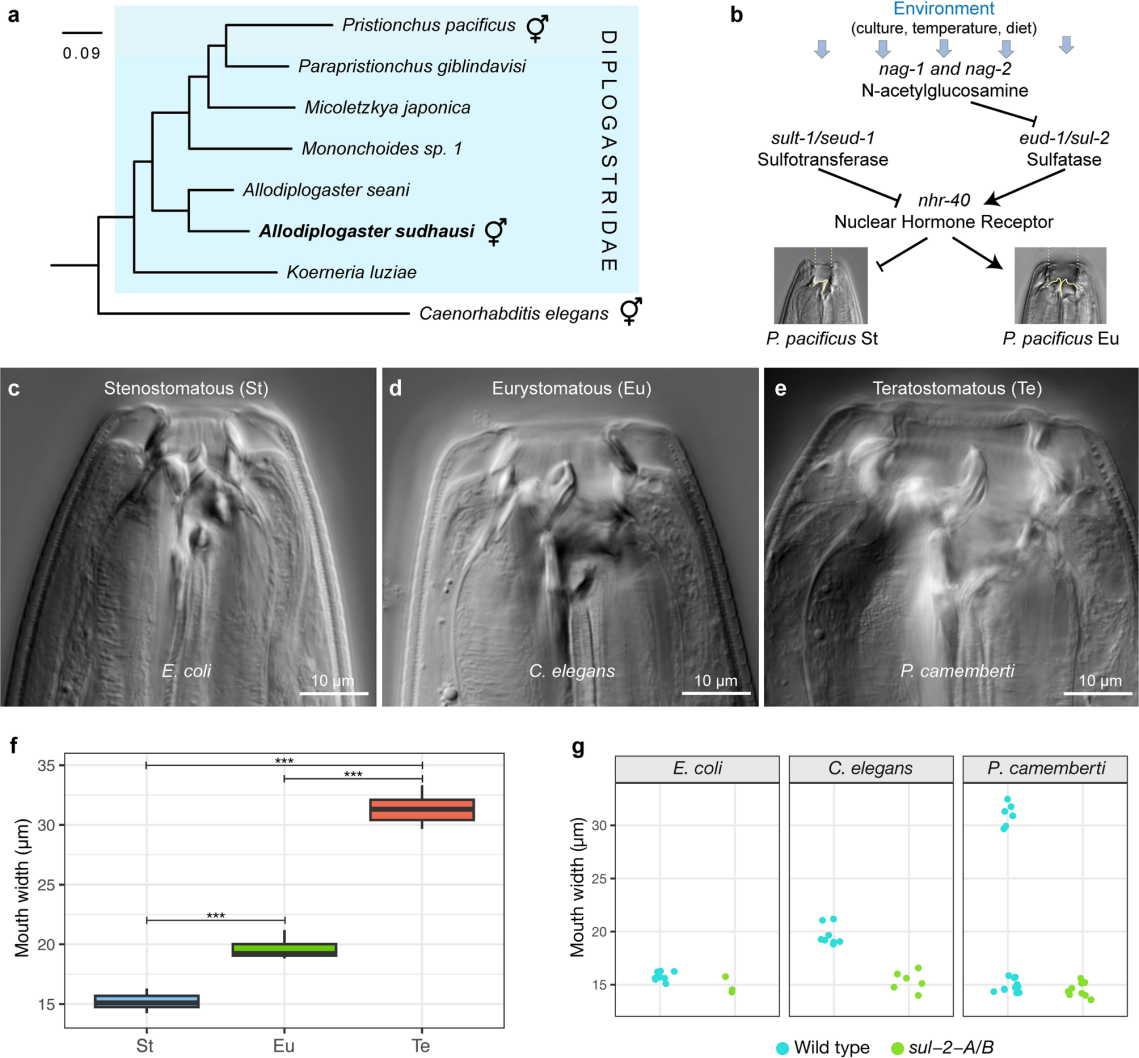
The nematode *Allodiplogaster sudhausi* can form three potential adult morphs, with conserved sulfatase genes regulating mouth-form phenotype. (**a**) *A. sudhausi* belongs to the Diplogastridae nematode family and is a distant relative of *Pristionchus pacificus*. Hermaphroditic symbols indicate species that are androdeiocious (hermaphrodite/male). The scale bar shows branch length. (**b**) The *P. pacificus* gene regulatory network controlling adult mouth-form phenotype has been well-elucidated, with many genes identified that act as a switch in becoming either Stenostomatous (St) or Eurystomatous (Eu). (**c**–**e**) *A. sudhausi* adults can form three possible mouth-form morphs, as indicated by differential interference contrast (DIC) microscopy pictures of (**c**) ‘narrow-mouthed’ St from an *E. coli* diet, (**d**) ‘wide-mouthed Eu’ from predation on *C. elegans* and (**e**) ‘monster-mouthed’ Teratostomatous (Te) from a fungal *P. camemberti* diet. (**f**) Mouth width measurements show there are significant differences between St, Eu and Te (Kruskal–Wallis, ***P < 0.001; pairwise Wilcox test, P < 0.01 for each comparison; n = 8 to 12 biological replicates). (**g**) Double mutant knock-outs in the sulfatases, *sul-2-A/B* remain St under all three diets, in stark contrast to wild type worms (n ≥ 3 biological replicates). (**f**,**g**) data obtained from previous work^18^.

Over time, genes present in the common ancestor of a taxon can diverge from an ancestral version in both sequence and function. Here, function may be a gene-intrinsic property related to expression or properties of gene product(s), or one related to shifts in the context in which those products act. Numerous studies have estimated the timing of divergence, often using substitution rates along with fossil records^1–3^. These are continuously being updated and optimised, enabling us to better understand the gradual changes in DNA and amino acid sequences. However, it is also vital to examine changes at the phenotypic (functional) level. Here, we compare two distantly related nematodes, *Pristionchus pacificus* and *Allodiplogaster sudhausi,* who nonetheless belong to the same superfamily, the Diplogastridae (Fig. 1a)^4^. Both these species display developmental (phenotypic) plasticity, the phenomenon whereby organisms with the same genotype can form different phenotypes based on environmental conditions. Recent work in the likes of spadefoot toads^5,6^, horned beetles^7^ and indeed diplogastrid nematodes^8^ has shown developmental plasticity plays a key yet under-appreciated role as a driver of evolutionary events and novelty. For instance, rapid evolutionary diversification in plastic feeding structure morphology and subsequent speciation occurred in the Diplogastridae, suggesting plasticity has strong adaptive value^9^. Similarly, evolution of increased numbers of ant worker castes through plasticity favours the division of labour and correlates with colony size^10^. Numerous other examples supporting the importance of developmental plasticity for evolution have recently been reviewed^11^.

In nematodes, Diplogastridae species display developmental plasticity in mouth-form phenotype, with most species capable of forming two alternative adult mouth-form morphs adapted to different environmental conditions^9^. For example, *P. pacificus* can form the narrow-mouthed stenostomatous (St) morph which lacks a sub-ventral tooth and is bacterial-feeding, or the wide-mouthed eurystomatous (Eu) morph which has two teeth and predates on other nematodes^12^. The mechanisms behind this plasticity have been well-studied, with important genes identified that regulate the two mouth-form phenotypes^13–17^ (Fig. 1b). We recently examined *A. sudhausi*, which diverged early in the Diplogastridae lineage and displays mouth-form plasticity. While *A. sudhausi* still retains the equivalent St and Eu morphs that are found in most diplogastrids, we were surprised to discover it can form three possible morphs (Fig. 1c–f). These morphs can be identified and distinguished by size and shape differences in their mouths. The newly identified third morph, termed teratostomatous (Te), has the widest mouth and appears when grown on the fungus *Penicillium camemberti*^18^. Interestingly, this Te morph, in contrast to St or Eu individuals, exhibits cannibalism against genetically identical kin, a new behaviour in nematodes. Taken together, both the *P. pacificus* and *A. sudhausi* mouth-forms occur as a polyphenism, i.e. produce discrete alternative phenotypes. This polyphenic trait enables us to compare discrete characteristics between species and is ideal to examine the phenotypic changes of ancestral genes over time.

Multiple genes involved in mouth-form regulation have already been identified in *P. pacificus*, based on previous studies that used forward and reverse genetics to generate knock-out mutants (Fig. 1b). Some mouth-form regulators act as developmental switches, with knock-out mutants resulting in the absence of one of the alternative forms, resulting either in an all-St or all-Eu phenotypes^13–16,19^. Therefore, these genes are ideal candidates to examine the divergence of homologs between both *P. pacificus* and *A. sudhausi*. Importantly, functional studies of homologs of *eud-1* were previously performed in *A. sudhausi*^18^. In that study, we found two *eud-1* paralogs*,* termed *sul-2-A* and *sul-2-B*. These sulfatase gene(s) act as a switch in both species and regulate the Eu morph, with knock-outs remaining St across all conditions (Fig. 1g). Notably, in *A. sudhausi* the two genes additionally regulate the third Te morph, indicating the additional morph may have evolved by co-option of the existing Eu machinery. Thus, the sulfatase genes display conservation of their function between both species in Eu regulation; however, they also evolved a novel role in Te regulation in *A. sudhausi*. We therefore wanted to examine other mouth-form genes to see if they show similar conservation between both species, or whether there is further novelty to be found.

Estimating divergence between *P. pacificus* and *A. sudhausi* is essential for understanding how long mouth-form genes have had to evolve independently. Amber fossils of diplogastrids date back to 99 million years ago (mya)^20^. Phylogenomic analysis, that incorporate both fossil dating and divergence estimates, suggest *A. sudhausi* diverged from its common ancestor with *P. pacificus* approximately 180 mya^21^. This is an exceedingly long period for evolutionary changes to occur, especially considering the short nematode generation times; four days for *P. pacificus*^22^ and eight days for *A. sudhausi*^23^.

The plasticity network in diplogastrid nematodes presents an ideal model for studying gene divergence through evolutionary time. Evaluating these two distantly related species offers insights into the evolution of plasticity. Genetic studies in *A. sudhausi* can shed light on the ancestry of the gene regulatory network (GRN) controlling mouth-form plasticity. For instance, it is thus far unknown whether the major regulators of plasticity are conserved over large evolutionary distances. Alternatively, these genes may undergo rapid turnover or retain sequence conservation while shifting away from a role in mouth-form. Given the many unknowns due to the absence of empirical studies, the comparison between *P. pacificus* and *A. sudhausi* can provide support for the long-held notion that developmental plasticity is a facilitator of novelty and, as such, subject to natural selection^24^. Overall, the retention of these genes and their role in the regulation of mouth-form plasticity over large evolutionary distances would strongly support their evolutionary importance in the regulation of mouth-form plasticity in the Diplogastridae.

In this study, we aim to examine the genetic machinery regulating mouth-form plasticity. Are the identified mouth-form control genes ancestral within the family or a recent acquisition in *Pristionchus*? We examined this by knocking out putative mouth-form genes in *A. sudhausi* to determine whether the role in mouth-form plasticity and manner of gene regulation is conserved in both *A. sudhausi* and *P. pacificus*. Our major goal was thus to examine whether mouth-form genes have an ancestral role; and, if so, to compare the phenotypic changes between both species and determine if there is divergence in the manner of gene regulation. This research is distinct as it encompasses a large time scale and focuses on functional genetic changes. Additionally, the presence of the third mouth-form in *A. sudhausi* enabled us to examine the role these genes may play in a novel trait. It is important to note that *A. sudhausi* recently underwent whole genome duplication (WGD)^25^, meaning that most genes have duplicate copies. However, due to the recency of the event, the majority of genes are redundant as there has been too little time for divergence between the copies. Therefore, two *A. sudhausi* genes needed to be targeted for every one homologous gene in *P. pacificus.* Crucially, the previous assembly and gene model annotation of *A. sudhausi* showed high completeness values and good coverage^25^, suggesting most genes can be correctly identified*.*

Here, we targeted six additional genes in *A. sudhausi,* and created combinations of mutant knock-out lines in order to compare the ways in which mouth-form genes showed conservation or divergence between *A. sudhausi* and *P. pacificus*. We found that knock-outs in these *A. sudhausi* homologous genes produced mutants with mouth phenotypes that differed from wild type and are thus involved in mouth-form regulation. However, the means in which genes controlled plastic traits often differed between species, with homologous genes displaying unique profiles, indicating distinct roles in the divergence of these genes from their ancestral forms.

## Results and discussion

**Fig. 2:**
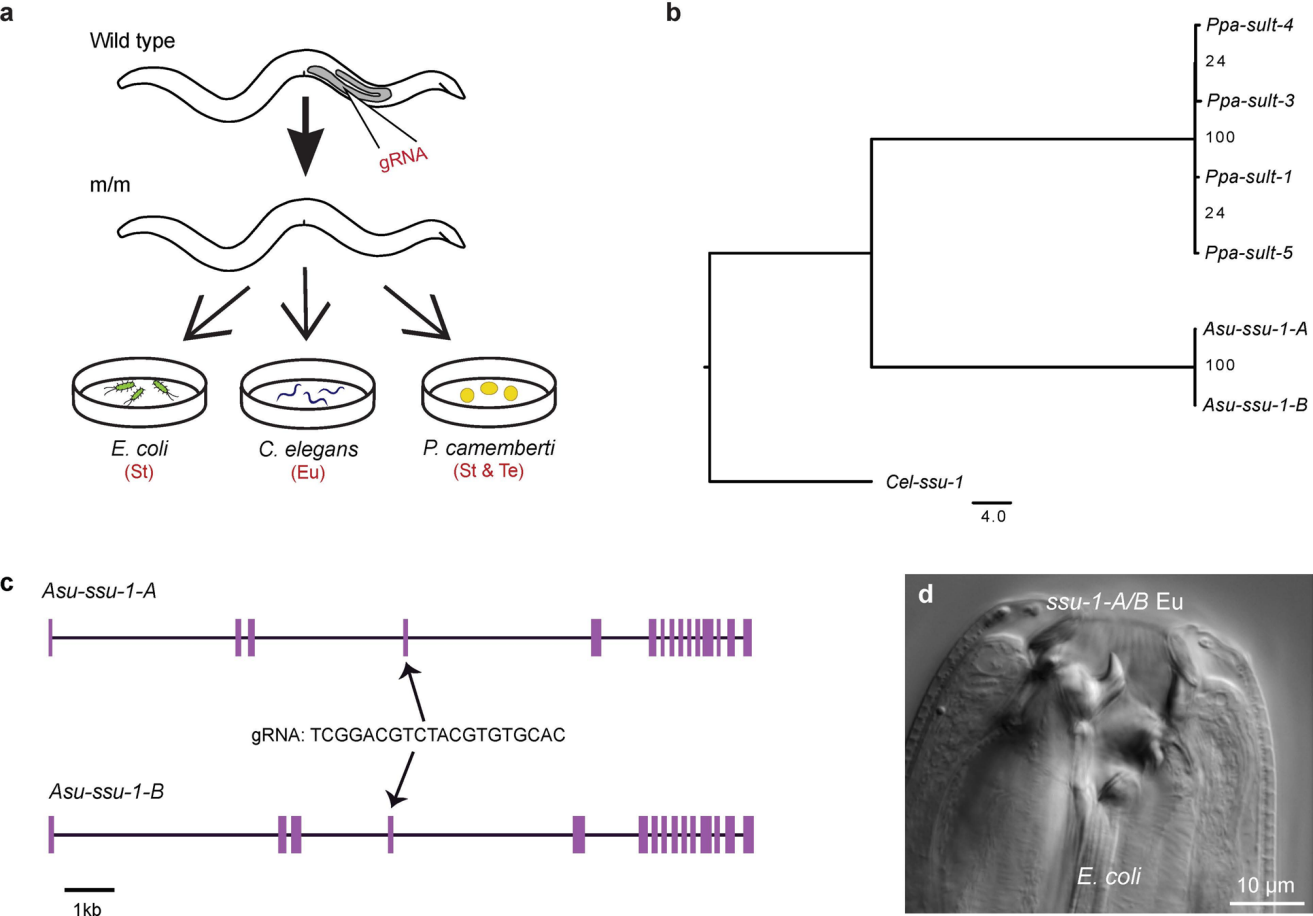
The sulfotransferase genes have a conserved role in Eu regulation. (**a**) Wild type worms were injected with a gRNA targeting both genes via CRISPR. Homozygous frameshift mutants (m/m) were selected for and grown on 3 diets with the following mouth-form in wild type adults: 1) *E. coli* bacteria that induces St, 2) *C. elegans* that induces Eu and 3) *P. camemberti* that induces either St or the novel Te morph. (**b**) The duplicate sulfotransferases *Asu-ssu-1-A* and *Asu-ssu-1-B* are homologous to four *P. pacificus* sulfotransferases, based on amino acid sequences, including *Ppa-sult-1* that has been shown to act as a switch in Eu regulation. Scale bar indicates branch length. (**c**) The predicted gene structure of *Asu-ssu-1-A* and *Asu-ssu-1-B* shows high similarity, as expected after WGD. A gRNA was designed that was able to target the predicted exon 4 in both genes. Scale bar: 1 kb. (**d**) DIC image of a *Asu-ssu-1-A/B* double mutant knock-out that becomes Eu instead of St on *E. coli*, which only produces the St morph in wild type worms.

We manipulated the mouth-form phenotype of *A. sudhausi* using three different diets (Wighard et al. 2024). In the wild type line, these diets have consistent and replicable phenotypes on: 1) *Escherichia coli,* which always becomes St, 2) *C. elegans,* which always becomes Eu and 3) *P. camemberti*, which becomes St or Te, depending on population density (Fig. 1g)^18^. This manipulation of diet allowed us to investigate the influence of putative mouth-form genes on the St, Eu and Te phenotypes. We used CRISPR to generate knock-out mutants of these genes of interest. A single guide RNA (gRNA) targeting each pair of WGD-derived genes was injected into wild type *A. sudhausi* worms to generate knock-out mutants (Fig. 2a). Homozygous frameshift mutations in the genes of interest were selected for in the subsequent progeny. Single gene knock-outs and double knock-outs of each pair of orthologous WGD-derived genes were generated. However, single mutants consistently displayed a wild type phenotype, meaning the double mutants were instead analyzed in-depth. These mutant lines were then grown up from eggs via a bleaching protocol^26^ on the three different diets and their adult phenotype was examined.

### Two sulfotransferase genes act as a switch in the regulation of the St morph

In *P. pacificus,* a cytosolic sulfotransferase-encoding gene, *sult-1* (also known as *seud-1*), homologous to *C. elegans ssu-1,* plays a key role as a switch gene. Knock-outs in *sult-1* prevent the narrow-mouthed St morph from forming, with mutants instead displaying the wide-mouthed Eu morph (Fig. 1b)^14,15^. This phenotype is thus the opposite of that seen in *P. pacificus* mutants of the sulfatase-encoding gene *eud-1*, which are all-St^13^. We previously showed the *A. sudhausi* sulfatase homologs of *P. pacificus eud-1,* termed *Asu-sul-2-A and Asu-sul-2-B,* together act as switch genes, with knock-out lines becoming St^18^ (Fig. 1g). We therefore wanted to determine if homologous *A. sudhausi* sulfotransferases also retain their switch gene function. Two homologs of *sult-1* had already been identified in *A. sudhausi*, *ssu-1-A* and *ssu-1-B* (Biddle and Ragsdale 2020) (Fig. 2b), which show high sequence similarity (Fig. 2c, Table S1) and are presumably duplicates resulting from the *A. sudhausi*-specific WGD^18^

We targeted *A. sudhausi ssu-1-A* and *ssu-1-B* and obtained single frameshift knock-out mutants using CRISPR; however, they displayed no mouth-form phenotype differences. This result was expected as previous studies already indicate most *A. sudhausi* duplicates resulting from WGD display redundancy^25^. We then crossed the single mutants together to obtain a double mutant and grew them on the three diets, as previously described. Strikingly, the *ssu-1-A/B* double mutant displayed an Eu phenotype on the St-inducing *E. coli* condition (Fig. 2d). This is similar to the phenotype of *P. pacificus sult-1* mutants which also remain Eu^14,15^. On *P. camemberti, ssu-1-A/B* was able to become Te (Fig. S1), but did not form the St morph whatsoever. Thus, the *ssu-1-A* and *ssu-1-B* genes appear to regulate the St phenotype. This finding indicates there is conservation of the role of the sulfotransferase genes in St regulation between both *A. sudhausi* and *P. pacificus.*

Note that the *ssu-1-A/B* double mutant line was noticeably unhealthy, with low survival. Specifically, *ssu-1-A/B* mutants had lower brood size (Fig. S2) and smaller body size (Fig. S3), with longer generation times and cannibalistic behaviour also observed. This meant very few worms survived bleaching, which is the standard way to obtain eggs on the three diets and prevent contamination. The *ssu-1-A/B* line was therefore not used for downstream mouth-form analysis. The sickly phenotype of the mutant line could be due to the negative effects of preventing sulfotransferase activity. Cytosolic sulfotransferases have a major role in detoxification as they promote excretion of xenobiotics^27^. Therefore, in the absence of these sulfotransferases, compounds can accumulate in cells and cause toxicity that produce such detrimental effects. In contrast, *P. pacificus* has multiple cytosolic sulfotransferases, meaning that functional redundancy among these enzymes may prevent *Ppa-sult-1* mutants from exhibiting the same detrimental phenotype (Fig. 2b)^28^.

### *nag* mutants display an intermediate mouth phenotype

**Fig. 3:**
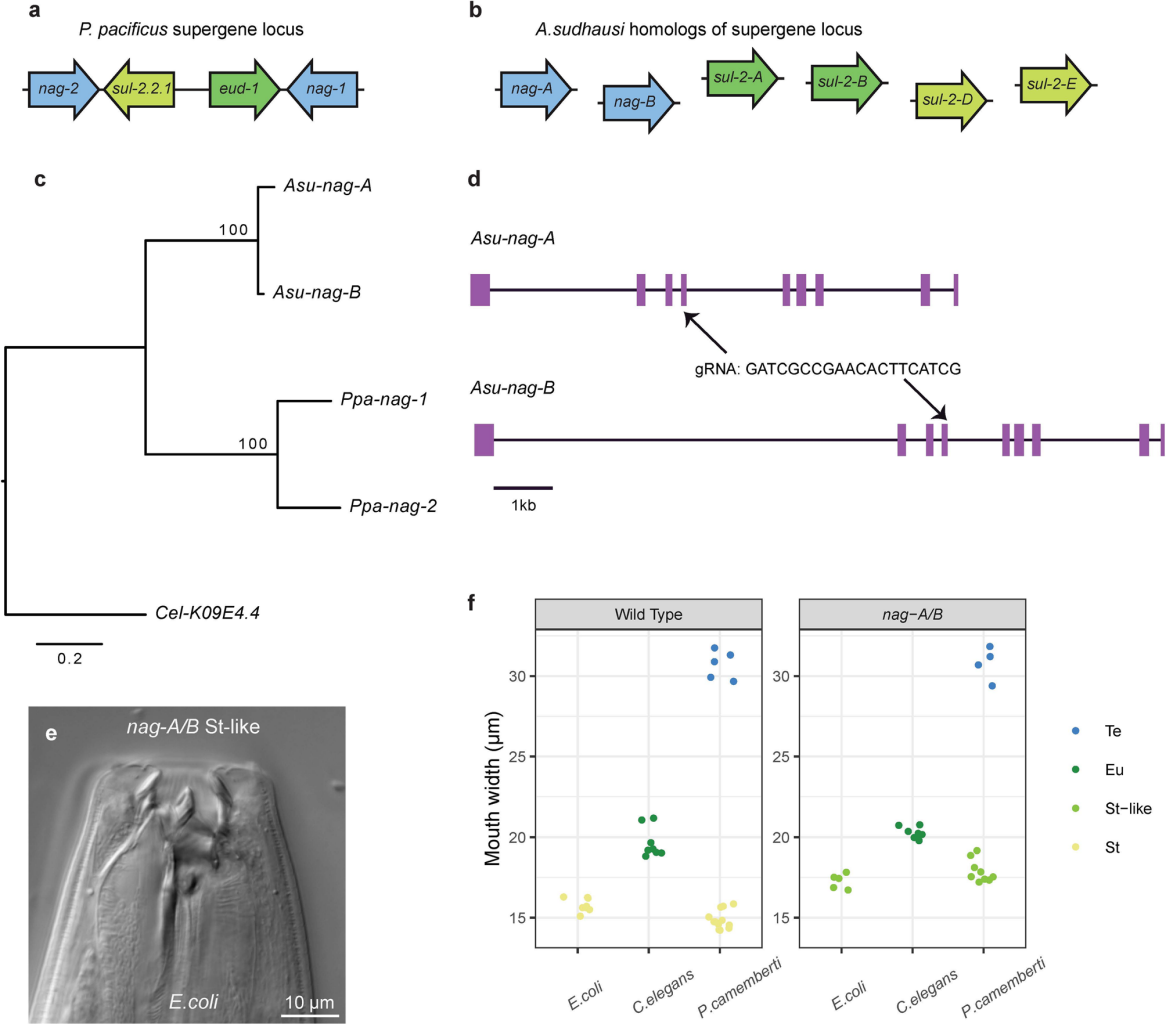
Knocking out *nag* genes produces an intermediate St phenotype. (**a**) The *P. pacificus* supergene locus contains the *nag-1* and *nag-2* genes in tandem orientation surrounding sulfatase encoding genes, including *eud-1* which is involved in mouth-form regulation and its gene duplicate *sul-2.2.1*, which plays no role in mouth-form. (**b**) *A. sudhausi* does not have a super gene locus. The homologs of those found in *P. pacificus* are found on separate contigs and shown. Colour coding matches the *A. sudhasui* genes with their *P. pacificus* homologs. (**c**) The two duplicate *A. sudhausi nag-A* and *nag-B* genes are homologous to the *P. pacificus nag* genes that underwent a local gene duplication after *A. sudhausi* and *P. pacificus* diverged. The *C. elegans* ortholog is used as an outgroup. Scale bar shows branch length. (**d**) The predicted gene structure is similar between *Asu-nag-A* and *Asu-nag-B*, with the same predicted number of exons. One gRNA was used to target exon 3 of both genes. (**e**) On *E. coli*, the *A. sudhausi nag-A/B* double mutant knock-out forms an intermediate ‘St-like’ phenotype whose mouth width is between wild type St and Eu. (**f**) The St-like morph is found on both *E. coli* and *P. camemberti* in *nag-A/B* mutants, in contrast to the wild type which becomes St (n ≥ 4 biological replicates). The narrow morph from *E. coli* and occasionally *P. camemberti* diets is referred as either ‘St’ or ‘St-like’ on the x axis, depending on its respective mouth width.

Next, we examined homologs of a recently-evolved supergene locus that acts as a major regulator of mouth-form plasticity in *P. pacificus*^16^ (Fig. 3a). This locus contains *eud-1* as well as two recently duplicated genes, *nag-1* and *nag-2* that encode N-acetylglucosaminidases. Knock-outs in both *nag* genes in *P. pacificus* led to constitutive expression of Eu, with the St morph no longer formed. These duplicates are in tandem orientation and located close to *eud-1*. Interestingly, the locus is not present in *A. sudhausi,* as it formed after the ancestor of *P. pacificus* and *A. sudhausi* diverged^29^ (Fig. 3b). We were compelled to examine whether the role of the ancestral *nag* gene in mouth-form regulation preceded or followed formation of the supergene locus. There are three possibilities: 1) the ancestral *nag* was already involved in mouth-form regulation before becoming part of a supergene locus, 2) the ancestral *nag* played no role in mouth-form regulation prior to its duplication, meaning it evolved this function along with becoming part of a supergene, or 3) the ancestral *nag* was not involved in mouth-form regulation but independently co-evolved this function in both *A. sudhausi* and *P. pacificus*; although this latter possibility is less likely it cannot be ruled out.

We identified two *nag* homologs in *A. sudhausi*, which we termed *nag-A* and *nag-B* based on previous nomenclature (Fig. 3c). As the *Pristionchus nag* duplication took place after its ancestor diverged from *Allodiplogaster*, the two *nag* genes in *A. sudhausi* would be WGD-derived duplicates of the single ancestral *nag* gene (Fig. 3d)*.* The *nag* duplication events in both species are thus independent from one another. We again found single mutants displayed a wild type phenotype across all diets, indicating redundancy in mouth-form function. Notably, the *nag-A/B* double mutant did not display the wild type St phenotype (Fig. 3e). Instead, *nag-A/B* displayed an intermediate ‘St-like’ phenotype, with the mouth width lying in-between that of wild type St and Eu morphs. Although the double mutant did not form the regular St phenotype on either *E. coli* or *P. camemberti,* it could still become Eu on *C. elegans* and Te on *P. camemberti* (Fig. 3f). This intermediate phenotype suggests *A. sudhausi nag-A* and *nag-B* genes together have quantitative effects. The phenotype in *A. sudhausi* thus differs from that in *P. pacificus*. In *P. pacificus*, the *nag* genes redundantly contribute to the switch, whereas in *A. sudhausi* they appear to be involved in the execution of the St morph and display a quantitative effect on mouth form. Importantly, the *nag* genes are involved in St regulation in both *A. sudhausi* and *P. pacificus*, suggesting *nag* genes have an ancestral role in formation of the St phenotype in Diplogastridae. Therefore, the involvement of the *nag* genes in mouth-form plasticity likely preceded formation of the supergene locus.

### *nhr-40* mutants produce an intermediate phenotype of the novel Te morph

**Fig. 4:**
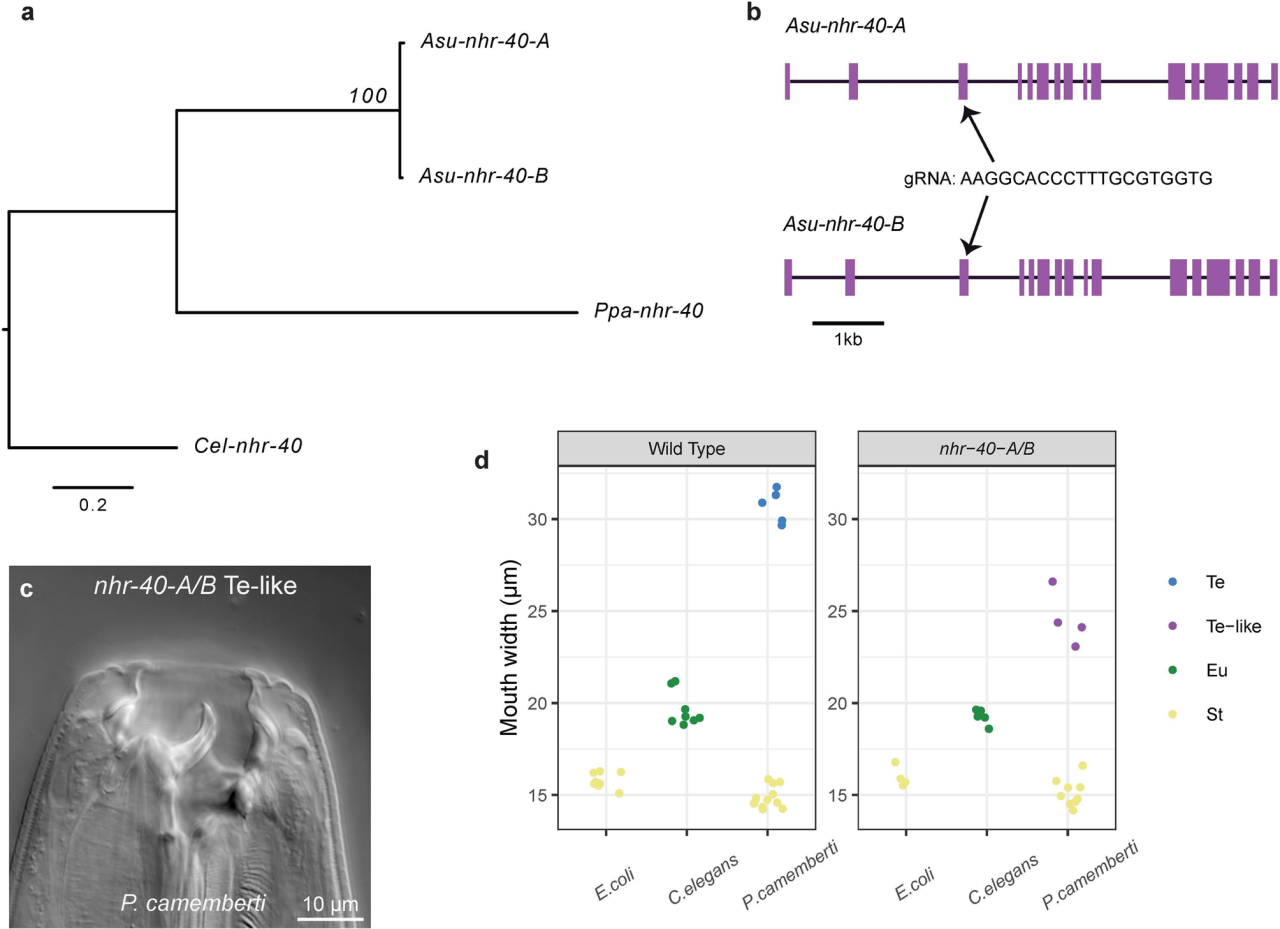
Knocking out *nhr-40* genes produces an intermediate Te phenotype. (**a**) *Asu-nhr-40-A* and *Asu-nhr-40-B* were identified as the homologs of *Ppa-nhr-40*, with *nhr-40* as an outgroup. (**b**) The gene structure is highly similar between the *A. sudhausi nhr-40* genes. A gRNA was designed that targeted exon 3 of both genes. (**c**) The *nhr-40-A/B* double mutant knock-out does not produce the regular Te phenotype on *P. camemberti*; instead, it forms a narrower ‘Te-like’ mouth that is otherwise similar in morphology. (**d**) The nhr-40-A/B ‘Te-like’ is noticeable narrower than wild type Te and falls in-between the Eu and Te mouth width (n ≥ 4 biological replicates). The largest mouth-form found on *P. camemberti* was referred to either as ‘Te’ or ‘Te-like’ depending on mouth width.

Comparisons between mouth-form genes in *A. sudhausi* and *P. pacificus* have thus far led to 1) conservation of function alone (*Ppa-sult-1, Asu-ssu-1-A/B*), 2) conservation of function and gain of novel function (*Ppa-eud-1, Asu-sul-2-A/B),* and 3) partial conservation of function, with retention of St regulation in both species, but only acting as a switch in *P. pacificus* (*Ppa-nag-1/2, Asu-nag-A/B*). Next, we examined the nuclear hormone receptor-encoding gene *nhr-40.* This gene acts as a switch in *P. pacificus,* with loss of function *nhr-40* mutants preventing the Eu morph from being formed^19^, similar to *Ppa-eud-1* mutants (Fig. 1b). We identified two WGD-derived homologous gene candidates in *A. sudhausi*, which we termed *nhr-40-A* and *nhr-40-B* (Fig. 4a). We again obtained frameshift knock-out mutant lines using CRISPR and examined their phenotypes on the three diets.

Like the other WGD genes, *Asu-nhr-40-A* and *Asu-nhr-40-B* show high similarity in gene sequence and structure (Fig. 4b, Table S1). The single knock-outs mutants again displayed a wild type phenotype, with gene redundancy occurring. However, the double mutant knock-out *nhr-40-A/B* displayed a clear yet unexpected mouth-form phenotype. The *nhr-40-A/B* knock-out line developed an intermediate ‘Te-like’ phenotype on the *P. camemberti* diet (Fig. 4c,d). That is, the mouth width lay in-between that of the Eu and Te phenotype, suggesting the genes have quantitative effects. In contrast, adults could still become St and Eu*,* as seen in wild type (Fig. 4d). Therefore, the *nhr-40-A* and *nhr-40-B* genes appear to be involved in regulating the novel Te phenotype. This is in stark contrast to *P. pacificus* where *nhr-40* acts as a switch gene in Eu regulation. Thus, the *nhr-40* genes in *A. sudhausi* firstly differ in that they are involved in regulation of a different mouth phenotype (Eu *vs.* Te) and, secondly, differ in that they do not act as a switch gene. Therefore, relative to other mouth-form genes studied here, there is high divergence in the way *nhr-40* genes regulate mouth-form formation between *A. sudhausi* and *P. pacificus*.

The findings of the *A. sudhausi nhr-40-A/B* mutant can be compared to the *nag-A/B* results. There is a similarity in that, in both cases, the *A. sudhausi* homologs do not function as a switch and instead appear to have quantitative effects. However, there is a marked difference in the targets of the genes; the *nag* genes regulate the St phenotype in both *A. sudhausi* and *P. pacificus*, while the *nhr-40* genes regulate Eu in *P. pacificus* and Te in *A. sudhausi*. This unexpected finding is beneficial in that it allows us to formulate hypotheses on the timing of a particular evolutionary novelty in *A. sudhausi*. We previously showed that both WGD and evolution of the novel Te morph occurred after *A. sudhausi* diverged from its closest known relatives^18^; however, the timing of the Te morph and WGD relative to each other could not then be determined. Here, we show the *A. sudhausi* WGD-derived *nhr-40* genes are involved in formation of the Te mouth-form. This suggests that the Te morph evolved before WGD, as it is unlikely the *nhr-40* genes would both independently evolve to regulate this new morph. Thus, the evolution of the third morph might have preceded WGD in the lineage leading to *A. sudhausi*.

### The *nag *and *nhr-40* genes function independently of one another

**Fig. 5:**
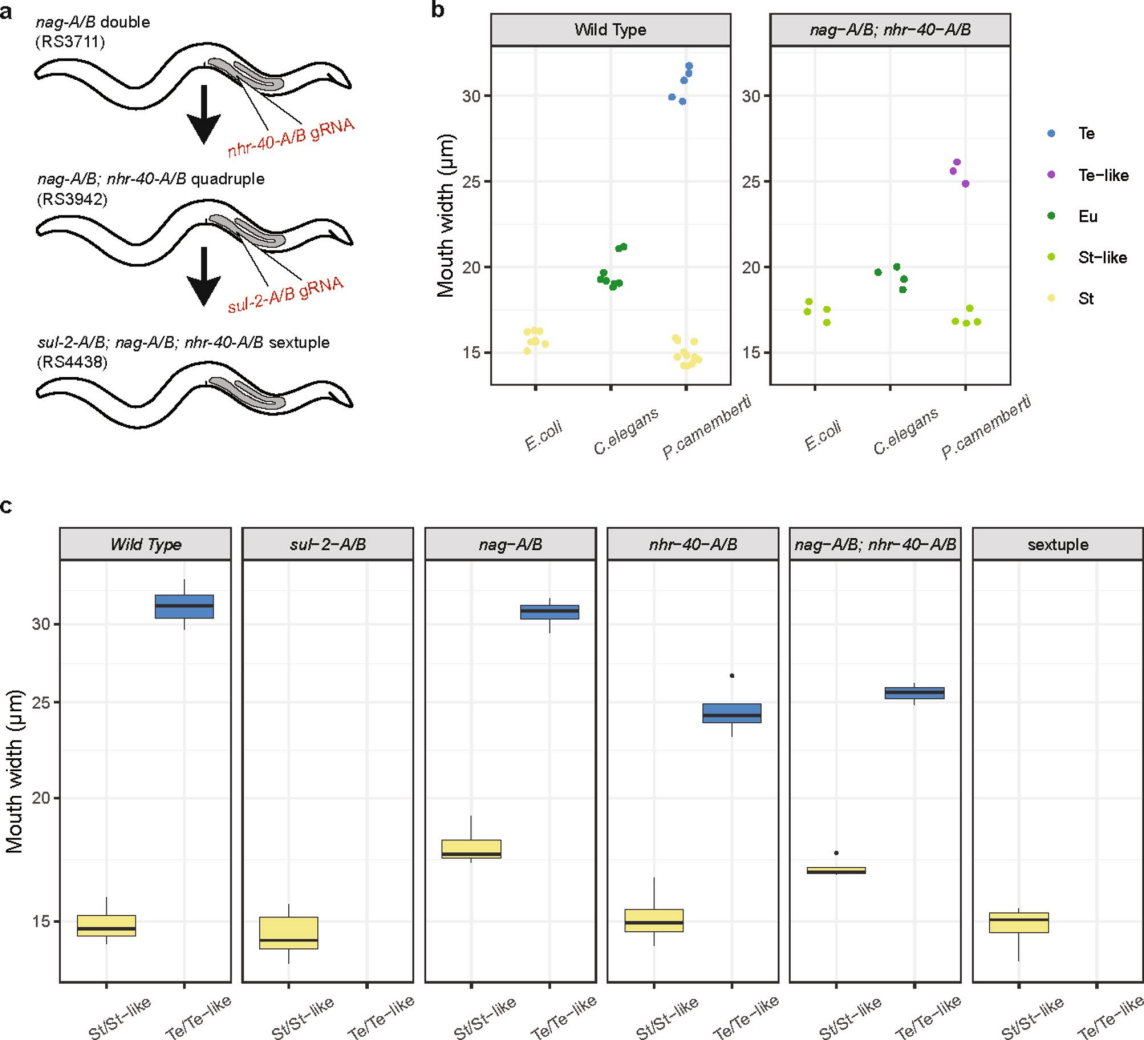
Combined mutations of mouth-form genes show epistasis in *A. sudhausi*. (**a**) The gRNA that targets both *nhr-40-A* and *nhr-40-B* was injected into the *A. sudhausi nag-A/B* double mutant line to obtain the *nhr-40/A/B; nag-A/B* quadruple mutant knock-out. The gRNA that targets *sul-2-A* and *sul-2-B* was then injected into the quadruple mutant to obtain the *sul-2-A/B; nag-A/B; nag-A/B* sextuple knock-out mutant. (**b**) The *nhr-40/A/B, nag-A/B* quadruple mutant knock-out showed both the ‘St-like’ phenotype of *nag-A/B* on St-inducing diets as well as the ‘Te-like’ phenotype of *nhr-40-A/B*, implying these gene pairs function independently of one another (n ≥ 3 biological replicates). (**c**) The mouth width of all examined mutant lines grown on *P. camemberti* is shown (n ≥ 3 biological replicates). This diet can produce either St or Te adults in wild type. The mutants are shown next to one-another for comparison. The sextuple mutant remained St, indicating *sul-2-A/B* is epistatic to both the *nag* and *nhr-40* genes in *A. sudhausi*.

Thus far, all homologous gene candidates vary in their degree of conservation of mouth-form function over large evolutionary distances (Table 1). This suggests there are multiple ways genes can functionally diverge. However, while the functional roles of genes have been compared between *A. sudhausi* and *P. pacificus*, the regulatory network and epistatic interactions of these genes had not been shown in *A. sudhausi.* We therefore generated combined *A. sudhausi* knock-out mutants. We first examined the relationship of *nhr-40-A/B* and *nag-A/B* genes to each other as their respective double mutant knock-outs resulted in intermediate mouth-forms (Figs. 3e,f, 4c,d). These mutants display contrasting phenotypes because they regulate different mouth-form morphs, making them ideal to study their genetic relationship to one another. For instance, it could be epistatic, where knocking out one gene pair masks the expression of the other, or alternatively, they could function independently of one another. We injected a gRNA targeting both *nhr-40-A* and *nhr-40-B* into the *nag-A/B* double mutant and obtained a *nhr-40/A/B, nag-A/B* quadruple mutant knock-out (Fig. 5a). Strikingly, this quadruple mutant displayed the phenotypes of both the *nag-A/B* and the *nhr-40-A/B* double mutant lines on different diets (Fig. 5b). Specifically, on St-inducing *E. coli*, the quadruple mutant formed the intermediate wider St phenotype that was also seen in *nag-A/B* on the same diet. Similarly, on Te-inducing *P. camemberti*, the quadruple mutant formed the intermediate narrow Te mouth that was seen in *nhr-40-A/B* on the *P. camemberti* diet. No wild type St or Te mouth-forms were observed whatsoever in the quadruple mutant. Therefore, knocking out all four genes did not affect the phenotype of the other double mutant. We thus conclude that the *nhr-40-A/B* and *nag-A/B* genes function independently of each other, in parallel pathways of the mouth-form GRN. This is in contrast with *P. pacificus*, where the *nag* genes are believed to lie upstream of *nhr-40*^16^.

**Table 1:** Homologous genes and their manner of conservation between *P. pacificus* and *A. sudhausi*. S: Switch genes; Q: Quantitative effects.

Homologous mouth-form genes in diplogastrids		Role in mouth-form plasticity		Manner in which gene is conserved of diverged	
*P. pacificus* gene name(s)	*A. sudhausi* gene names	Role in *P. pacificus*	Role in *A. sudhausi*	Conservation	Divergence
*eud-1*	*sul-2-A &* *sul-2-B*	Regulate Eu (S)	Regulate Eu (S) Regulate Te (S)	Switch gene in Eu regulation	Novel function as switch gene in to morph
*sult-1/seud-1*	*ssu-1-A &* *ssu-1-B*	Regulate St (S)	Regulate St (S)	Switch gene in St regulation	/
*nag-1 &* *nag-2*	*nag-A &* *nag-B*	Regulate St (S)	Regulate St (Q)	Regulation of St morph	Switch genes in *P. pacificus*; additive effects in *A. sudhausi*
*nhr-40*	*nhr-40-A &* *nhr-40-B*	Regulate Eu (S)	Regulate Te (Q)	/	Novel function in Te regulation

### The sulfatase genes act downstream of *nhr-40 *and *nag* genes

Lastly, we examined the relationship of the sulfatase switch genes to *nhr-40* and *nag* genes. *Asu-sul-2-A/B* mutants are known to be constitutively St under all conditions^18^. We injected the *sul-2-A/B* gRNA into the *nhr-40-A/B; nag-A/B* quadruple mutant line to obtain a *sul-2-A/B*; *nhr-40/A/B; nag-A/B* sextuple mutant (Fig. 5a). Then, we grew the sextuple mutant line on St-inducing *E. coli*. On this diet, *nag-A/B* forms an intermediate wider St mouth, while *sul-2-A/B* forms the regular St mouth-form. Notably, the sextuple mutant line displayed the regular St phenotype (Fig. S4; Table S2), indicating the sulfatase genes are epistatic to the *nag* genes. Interestingly, this is also the case in *P. pacificus*, showing conservation of the gene regulatory pathway between both species.

We then grew the sextuple mutant on Te-inducing *P. camemberti*. The sulfatase double mutant is consistently St on all conditions; while the *nhr-40* mutant can form the narrower Te-like morph. Strikingly, we found the *A. sudhausi* sextuple mutant did not become Te at all (Fig. 5c; Fig S3; Table S2), indicating *sul-2-A/B* acts downstream of *nhr-40-A/B*. Indeed, the sextuple mutant remains St under all conditions. Therefore, the sulfatase genes act downstream to the other mouth-form genes studied. This order in the GRN contrasts with the hypothesis in *P. pacificus* that *nhr-40-A/B* is epistatic to *sul-2-A/B*. While knock-outs of the homologs in *P. pacificus* have the same phenotype, epistatic interactions are harder to interpret; although *Ppa-nhr-40 gain-of-function eud-1* alleles may suggest both genes act in parallel to each other^30^. Overall, the findings in *A. sudhausi* provide insight into the GRN in diplogastrids and its epistatic interactions.

## Conclusion

In this work, we examined homologous genes (based on sequence conservation) between two highly diverged nematode species. Remarkably, all candidate genes retained a role in mouth-form regulation in both *A. sudhausi* and *P. pacificus*. However, the exact role of these genes was not always conserved and, instead, displayed distinct identities of functional diversification in both species (Table 1). All gRNAs were targeted to early exons to limit the likelihood of frameshift mutants producing functional - if short - proteins; however, we cannot completely rule out this possibility, and future work would be necessary to examine this. Overall, our results indicate there are multiple ways genes can functionally diverge over time, suggesting the complex mouth-form GRN is subject to continued selection at the gene-specific level. Indeed, the differences in the means of conservation may be due to relaxed pressure that allowed different pathways or parts of the GRN to evolve distinctively. Nevertheless, selection for mouth-form plasticity must be strong as the same homologous genes are involved in mouth-form regulation despite the two species last sharing a common ancestor nearly 200 million years ago^21^. These findings suggest plasticity plays a critical in the survival and ongoing evolution of diplogastrid worms. Our results parallel recent work in pea aphids^31^- where duplicate genes regulate a morphological dimorphic trait—further supporting the idea that plasticity is a major driver of evolutionary novelty^24^.

While each of the homologous gene pairs in *A. sudhausi* showed different degrees of conservation, it is particularly interesting that only the genes encoding the sulfatases and sulfotransferases retain their role as switch genes across both species. The sulfation pathway has previously been identified as a key mouth-form regulator in *P. pacificus* from independent experiments including genetic and epigenetic studies, as well as natural variation studies through recombinant inbred line and quantitative trait locus (QTL) analysis^13,32^. Its additional importance in *A. sudhausi* suggests the sulfation pathway is crucial for regulating mouth-form plasticity across the Diplogastridae family. Nonetheless, further work is necessary to elucidate this pathway and better understand how and why sulfation processes are involved in nematode mouth-form plasticity^28^. Work is ongoing to identify the target molecules of sulfation processes, *i.e.* hormones or other signaling molecules.

Interestingly, in examining conservation of the *nhr-40* homologs, we can provide initial hypotheses regarding the previously held mystery of the relative timing of major evolutionary phenomena in *A. sudhausi*. Originally, we identified the evolution of both the novel Te morph and the incidence of WGD^18^; however, the chronology of these events could not be determined due to a sufficient lack of closely-related strains. Here, we suggest that the Te morph evolved before WGD occurred, as the *Asu-nhr-40-A/B* mutant did not form the wild type Te phenotype (Fig. 4c,d). It is highly unlikely these gene would independently gain the same novel function in parallel. Therefore, since both *nhr-40* genes display the same function, it suggests WGD should have taken place after gain of the Te morph. Alternatively, the critical event for the evolution of the Te morph required the evolution of new target gene(s) after WGD. For instance, a potential scenario is that a new binding site for NHR-40 evolved, meaning there is instead a novel set of target genes that produced this functional shift. Thus, the results obtained in this study allow the formulation of two alternative hypotheses that can be further addressed in future studies. Note that WGD was estimated to have taken place only 1.3 to 3.3 million mya^25^, meaning that the evolution of the Te morph happened in the time scale of a few million years.

In summary, our study strongly supports the idea that the identified mouth-form genes have ancestral functions in feeding structure plasticity throughout the Diplogastridae, but their specific roles have diverged in different lineages. While convergent evolution cannot be completely ruled out, it is extremely unlikely the four ancestral genes, *sul-2-A/B, ssu-1-A/B, nag-A/B* and *nhr-40-A/B,* would independently evolve a mouth-form phenotype. Therefore, the principle of parsimony suggests an ancestral function in mouth-form regulation. Note that although *A. sudhausi* may be seen as the more ‘ancestral’ species due to its phylogenetic position, both species are identical in evolutionary age, meaning the species most similar to the common ancestor cannot be determined with certainty unless these genes are investigated in a broader range of diplogastrids. Taken together, our study highlights the dynamic nature of gene divergence over time, adding to the body of work on sequence-level evolutionary changes.

## Materials and methods

### Nematode maintenance and inbreeding

The *A. sudhausi* inbred wild type strain SB413B/RS6132B^25^ and RS398 (*sul-2-A/B* double mutant knock-out)^18^ line was used. Further strains were generated via CRISPR knock-out engineering (described in detail further below): RS3711 (*nag-A/B* double mutant knock-out), RS3723 (*nhr-40-A/B* double mutant knock-out), RS3942 (*nag-A/B; nhr-40-A/B* quadruple mutant knock-out), RS4438 (*sul-2-A/B; nag-A/B; nhr-40-A/B* sextuple mutant knock-out). *C. elegans* (N2) was used as an Eu-inducing diet. All strains are frozen and kept at the Max Planck Institute for Biology. All nematodes were maintained on nematode growth medium (NGM) agar plates with *E. coli* OP50 bacteria.

### Nematode diets

Freshly starved NGM *E. coli* plates containing many eggs were bleached^26^ onto separate NGM containing three different food sources: 1) *E. coli* OP50*,* 2) *C. elegans* N2 and 3) *P. camemberti,* as previously described^18^. For *C. elegans,* plates with many larvae were washed with M9 and passed through a 20 µm nylon net filter (Merck) onto unseeded 6 cm NGM plates, so that only larvae passed through. *P. camemberti* was obtained from the Leipniz Institute DSMZ-German Collection of Microorganisms and Cell Cultures GmbH (https://www.dsmz.de) and maintained weekly on NGM plates. All assays were otherwise were maintained at 20˚C. For all experiments, only young hermaphrodites were selected (containing less than five eggs) for mouth-form identification and measuring.

### Phylogenetic trees and sequence analysis

The species tree was based on previous data^9^. For the gene trees, *P. pacificus* homologs were identified using sequence annotations from pristionchus.org (El Paco V3 2020). The corresponding protein sequences were BLASTed on wormbase.org (version WS284) to get *C. elegans* homologs. The predicted *A. sudhausi* protein sequences were identified on pristionchus.org using the previously published gene models^25^. The exact gene nomenclature and their corresponding annotations can be found in Table S3. The phylogenetic tree was generated using RAxML version 8.2.12 (raxmlHPC -f a -m PROTGAMMAAUTO -p 12345 -× 12345 -N 100), with maximum likelihood boostrap values included (raxmlHPC -f b -m PROTGAMMAILG)^33^. The phylogeny was then displayed using FigTree software (v.1.4.4) (tree.bio.ed.ac.uk/software/figtree). The amino acid sequences of all predicted proteins can be found in Data S1. To determine sequence similarity (Table S1), these sequences were compared on pristionchus.org/blast using ‘Allodiplogaster sudhausi proteins,^25^’ as the database.

### Microscopy images and mouth measurements

Young adult hermaphrodites were fixed onto a solution with 5% Noble Agar and 0.3% NaN3, with M9 buffer for resuspension. They were imaged at 100 × magnification using the Differential Interference Contrast (DIC) setting. Only worms fixed in the correct orientation (on their lateral side) were picked, as those not correctly oriented would vary in mouth width and add noise to the results, reducing the replicability of data values. Due to this, the number of technical replicates per biological replicated varied based on how many worms happened to be appropriately oriented for each assay. Z-stacks were taken of the head region and stored as raw data .czi files. Fiji/ImageJ^34^ was used to measure the width of the mouth (stoma), using the base of the cheilostom and the start of the gymnostom as the markers. All mouth measurements from a single experiment on a particular diet was counted as one biological replicate. The exception to this is when the diets produced different mouth-forms in one assay: Mouth-form was then used as an added grouping factor when analysing the data. The reason for this, is that the bimodality of the mouth-width on these diets would have given misleading results when calculating mean values. At least three biological replicates were used for each diet and corresponding mouth-form per strain. The exact measurements, number of replicates and experimental dates is shown in Data S2. For plotting, either box plots or dot plots were used. Box plots were preferentially used, but dot plots were used when data was bimodal. This is because the values obtained from a bimodal distribution cannot be accurately visualized with a box plot which covers the full range. Dot plots were created on ggplot2 using geom_jitter (with height importantly set to 0) with each biological replicate represented by an individual point. In order to represent data accurately, it was grouped by diet, biological replicate and mouth-form (the latter only in the case of bimodal data). Mouth-form rankings in the *P. camemberti* diet were based on previous results^18^, with St falling between 13–17.5 μm, Eu between 17.5–23 μm and Te between 28–34 μm. Date outside of these values were then phenotyped in mouth-form categories for the purposed of data visualization.

### Mouth-form phenotyping

Worms were counted and phenotyped under a Zeiss Stemi 508 light microscope when they reached adulthood. The mouth-form of adult worms was then determined from all three diets.

### Brood size and worm size measurements

To compare wild type and *ssu-1-A/B* double mutants, both brood size and worm length were measured. For brood size, young virgin hermaphrodites were moved from well-fed *E. coli* plates to NGM plates containing 30 µl of *E. coli*. They were transferred to fresh plates ever two days for a total of eight days. The number of live offspring that each hermaphrodite produced was counted. All plates were stored at 20 °C. For worm sizing, young adult hermaphrodites from *E. coli* were moved to NGM plates without bacteria. Bright-field images were taken at 20X magnification using the Axio Zoom V16 microscope. Analysis was done on Fiji/ImageJ using the Wormsizer plugin for ImageJ/Fiji^35^ to detect worm length.

### CRISPR injections to obtain mutants

CRISPR knock-outs were generated as previously described^25^. CRISPR RNAs (crRNAs) and trans-activating crispr RNA (tracrRNA) (Cat. No. 1072534) were synthesized by Integrated DNA Technologies (IDT), while the Cas9 endonuclease (Cat. No. 1081058) was purchased from IDT. The CRISPR/Cas9 complex was prepared by mixing 0.5 mg/ml Cas9 nuclease, 0.1 mg/ml tracrRNA, and 0.056 mg/ml crRNA in the TE buffer followed by a 10-min incubation at 37° C. Microinjections were performed in late-stage J4 hermaphrodites using an Eppendorf microinjection system. A single guide sequence was designed to target the two gene duplicates for each homologous gene pair of interest. Conserved regions were therefore targeted. Specific primers were designed for each individual gene in *A. sudhausi* (Table S4). Polymerase chain reactions (PCRs) were run using these primers on the F1 of injected worms. Heterozygotes were identified via Sanger sequencing (GENEWIZ Germany GmbH) and homozygous mutants were generated by selfing heterozygotes. Frameshift mutants were obtained for all strains (Table S5). For *A. sudhausi ssu-1* homologs, single mutants were obtained from the first CRISPR, which were then crossed to get the *ssu-1-A/B* double mutant. Single mutant *nag-A* and *nag-B* were also gained from one CRISPR injection, and then a second injection was performed to get the *nag-A-B* double mutant. The *nhr-40-A/B* double mutant line was obtained after a single injection, as were independent single *nhr-40-A & -B* mutants. The *nhr-40-A/B* gRNA was injected into the *nag-A/B* double mutant line and a quadruple mutant was found after one injection. The *sul-2-A/B* gRNA^18^ was injected into the *nag-A/B; nag-A/B* quadruple mutant line, which resulted in the *sul-2-A/B; nag-A/B; nag-A/B* sextuple mutant after a single injection. Exact strain designations are shown above.

## Supplementary Information

Supplementary Information 1.

Supplementary Information 2.

Supplementary Information 3.

Supplementary Information 4.

Supplementary Information 5.

Supplementary Information 6.

Supplementary Information 7.

Supplementary Information 8.

Supplementary Information 9.

## Data Availability

Strains are available upon request from the lead author (ralf.sommer@tuebingen.mpg.de). The authors affirm that all data necessary for confirming the conclusions of the article are present within the article, figures, and tables.
