# Supplementary figures and images for: Functional divergence of conserved developmental plasticity genes between two distantly related nematodes
Source: Sci Rep. 2025 Aug 5;15:28518. doi: 10.1038/s41598-025-14207-5 (PMC12325724; doi:10.1038/s41598-025-14207-5)

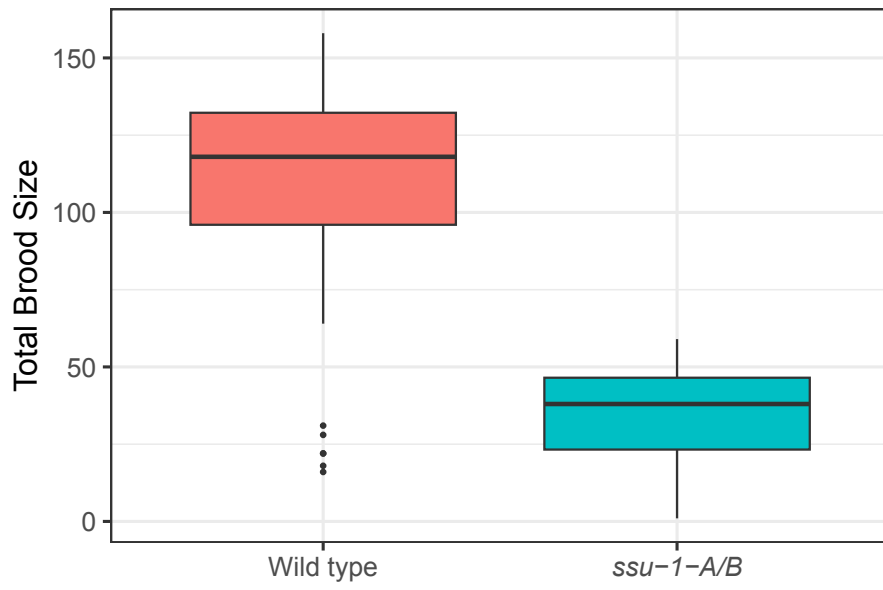

**Figure S2:** The *ssu-1-A/B* double mutant has a drastically lower brood size compared to wild type worms.

Supplement: Supplementary file 1 — Supplementary Information 1. [file 41598_2025_14207_MOESM1_ESM.pdf]

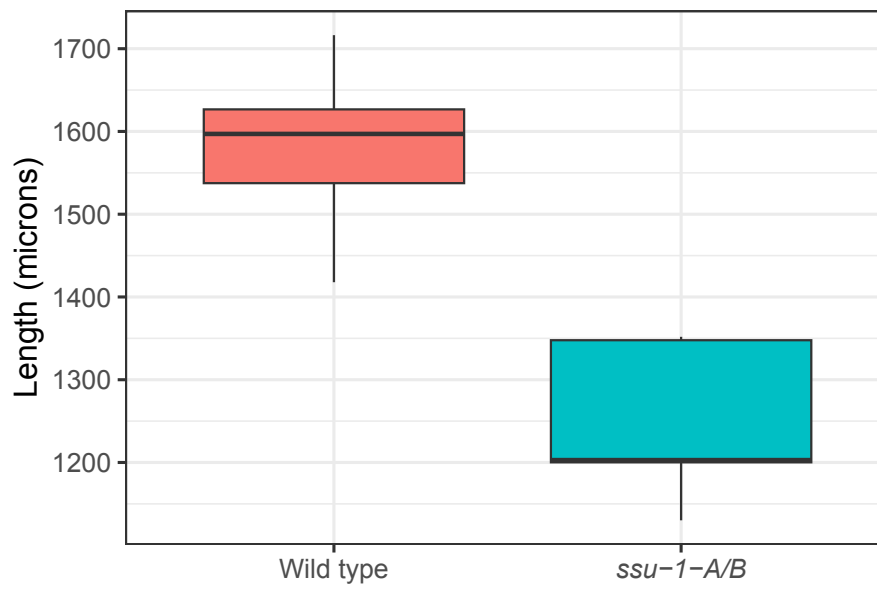

**Figure S3:** The *ssu-1-A/B* double mutant has a notably smaller body length compared to wild type worms.

Supplement: Supplementary file 2 — Supplementary Information 2. [file 41598_2025_14207_MOESM2_ESM.pdf]
