# Supplementary material for: Functional divergence of conserved developmental plasticity genes between two distantly related nematodes
Source: Sci Rep. 2025 Aug 5;15:28518. doi: 10.1038/s41598-025-14207-5 (PMC12325724; doi:10.1038/s41598-025-14207-5)
Supplement: Supplementary file 3 — Supplementary Information 3. [file 41598_2025_14207_MOESM3_ESM.pdf]

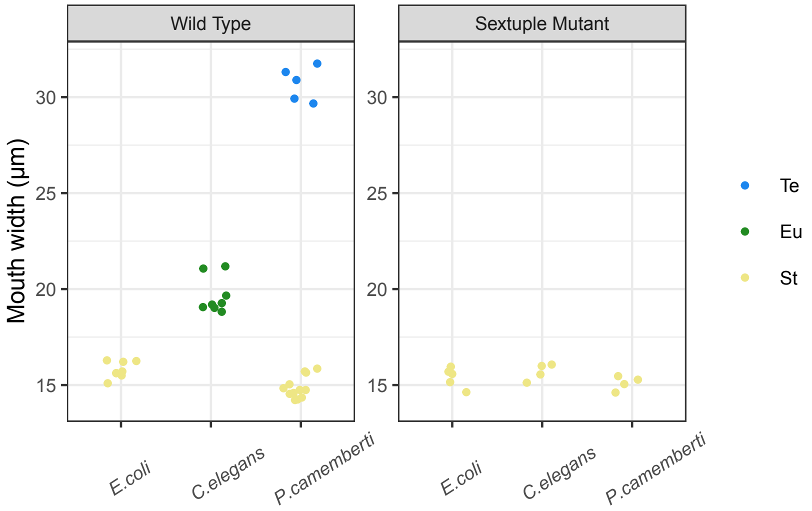

Figure S4: The *sul-2-A/B*; *nhr-40/A/B; nag-A/B* sextuple mutant remains St on all three diets and display a narrow mouth width, in contrast to wild type worms which become Eu on *C. elegans* and can become Te on *P. camemberti.*
