# Supplementary material for: Functional divergence of conserved developmental plasticity genes between two distantly related nematodes
Source: Sci Rep. 2025 Aug 5;15:28518. doi: 10.1038/s41598-025-14207-5 (PMC12325724; doi:10.1038/s41598-025-14207-5)
Supplement: Supplementary file 4 — Supplementary Information 4. [file 41598_2025_14207_MOESM4_ESM.pdf]

Table S1: The amino acid sequence identity shared between duplicate gene pairs in *A. sudhausi* is shown, with all having high overall similarity.

Gene pair	Sequence identity (%)
Asu-sul-2-A & -B	97
Asu-ssu-1-A & -B	94
Asu-nag-A & -B	90
Asu_nhr-40-A & -B	98
