# Supplementary material for: Functional divergence of conserved developmental plasticity genes between two distantly related nematodes
Source: Sci Rep. 2025 Aug 5;15:28518. doi: 10.1038/s41598-025-14207-5 (PMC12325724; doi:10.1038/s41598-025-14207-5)
Supplement: Supplementary file 5 — Supplementary Information 5. [file 41598_2025_14207_MOESM5_ESM.pdf]

Table S2: Phenotyping of the *sul-2-A/B*; *nhr-40/A/B; nag-A/B* sextuple mutant adult mouth-form on *C. elegans and P. camemberti* diets show they are consistently St.

Diet	Replicate	No. St	No. Eu	No. Te	Total no. worms
C. elegans	1	6	0	0	6
C. elegans	2	4	0	0	4
C. elegans	3	11	0	0	11
C. elegans	4	12	0	0	12
P. camemberti	1	6	0	0	6
P. camemberti	2	8	0	0	8
P. camemberti	3	33	0	0	33
P. camemberti	4	25	0	0	33
P. camemberti	5	55	0	0	55
P. camemberti	6	82	0	0	82
P. camemberti	7	66	0	0	66
