# Supplementary material for: Functional divergence of conserved developmental plasticity genes between two distantly related nematodes
Source: Sci Rep. 2025 Aug 5;15:28518. doi: 10.1038/s41598-025-14207-5 (PMC12325724; doi:10.1038/s41598-025-14207-5)
Supplement: Supplementary file 6 — Supplementary Information 6. [file 41598_2025_14207_MOESM6_ESM.pdf]

Table S3: The gene names and respective annotations of the amino acid sequences, which can be found on pristionchus.org for *A. sudhausi* (Wighard *et al.,* 2022) and *P. pacificus* (El Paco annotation v3, 2020).

Species	Gene name	Annotation
A. sudhausi	*Asu-nag-A*	ALDISUDHAUS000014281
A. sudhausi	*Asu-nag-B*	ALDISUDHAUS000017088
A. sudhausi	*Asu_nhr-40-A*	ALDISUDHAUS000009481
A. sudhausi	*Asu_nhr-40-B*	ALDISUDHAUS000005589
A. sudhausi	*Asu-ssu-1-A*	ALDISUDHAUS000003619
A. sudhausi	*Asu-ssu-1-B*	ALDISUDHAUS000003344
P. pacificus	*Ppa-nag-1*	PPA06134
P. pacificus	*Ppa-nag-2*	PPA34489
P. pacificus	*Ppa-nhr-40*	ppa_stranded_DN28158_c0_g3_i3
P. pacificus	*Ppa-sult-1/seud-1*	PPA12547
P. pacificus	*Ppa-sult-3*	PPA06620
P. pacificus	*Ppa-sult-4*	PPA22156
P. pacificus	*Ppa-sult-5*	PPA41942
