# Supplementary material for: Functional divergence of conserved developmental plasticity genes between two distantly related nematodes
Source: Sci Rep. 2025 Aug 5;15:28518. doi: 10.1038/s41598-025-14207-5 (PMC12325724; doi:10.1038/s41598-025-14207-5)
Supplement: Supplementary file 7 — Supplementary Information 7. [file 41598_2025_14207_MOESM7_ESM.pdf]

Table S4: The primer sequences to amplify the specific gene duplicates and identify mutants after CRISPR injections.

Primer name	Primer sequence (5' - 3')	Forward/Reverse	Target
sul-2-A_F	AGAATGTAGCCAGGCAAGC	Forward	*Asu-sul-2-A*
sul-2-A_R	CTCAGTCGACATGGAAAAGC	Reverse	*Asu-sul-2-A*
sul-2-B_F	ATTGCAGGATACGGCGACC	Forward	*Asu-sul-2-B*
sul-2-B_R	CTAATCTCGTCTATGCCGACG	Reverse	*Asu-sul-2-B*
ssu-1-A_F	TCTTCTTGCGACCAATGCGG	Forward	*Asu-ssu-1-A*
ssu-1-A_R	TAGAGCAGTCTGGACAAAGC	Reverse	*Asu-ssu-1-A*
ssu-1-B_F	TGATTGCGCGAAACGGAGAC	Forward	*Asu-ssu-1-B*
ssu-1-B_R	ATTAGAAGGATTGGCCGTGC	Reverse	*Asu-ssu-1-B*
nag-A_F	ATGGACTCGGTTACTTCACC	Forward	*Asu-nag-A*
nag-A_R	GACATTTGCTGGCTTCGTGC	Reverse	*Asu-nag-A*
nag-B_F	CAGTTCCACCAAACCATCG	Forward	*Asu-nag-B*
nag-B_R	TTGCTCCACCTCAATTTACG	Reverse	*Asu-nag-B*
nhr-40-A_F	CCTTAAATTAGGCTTTGAGC	Forward	*Asu-nhr-40-A*
nhr-40-A_R	CACTCTCCATTACCATACACT	Reverse	*Asu-nhr-40-A*
nhr-40-B_F	CTTGCGTAACTCCTTCTAATC	Forward	*Asu-nhr-40-B*
nhr-40-B_R	AGTATTGAGGTGAAGCTGGC	Reverse	*Asu-nhr-40-B*
