# Supplementary material for: Functional divergence of conserved developmental plasticity genes between two distantly related nematodes
Source: Sci Rep. 2025 Aug 5;15:28518. doi: 10.1038/s41598-025-14207-5 (PMC12325724; doi:10.1038/s41598-025-14207-5)
Supplement: Supplementary file 8 — Supplementary Information 8. [file 41598_2025_14207_MOESM8_ESM.pdf]

Table S5: The mutant knock-outs, with their respective allele and strain names, as well as resulting genotypes and phenotypes. Deletion is indicated by a hyphen (-) in the sequence. 'Del' stands for deletion and 'Ins' stands for insertion.

***A. sudhausi* knock-outs**	**Strain**	**Allele**	**Mutation**		**Mutation in Sequence**		**Phenotype**
			**A**	**B**	**A**	**B**	
nag-A	RS3710	tu1497	7bp del	/	CGTCCAGAT-------CACTTCATC		WT
nag-B	RS3733	tu1472	/	8bp del		CCGCTCTT--------TCGCCGAA	WT
nag-A/B	RS3711	tu1499 (A) & tu1472 (B)	8bp ins	8bp del	TCGTCCAGAACACTTCATCGCCGAA	CCGCTCTT--------TCGCCGAA	St-like instead of St
nhr-40-A	RS3722	tu1509	11bp del	/	AAAGGGTGC-----------TTCTCGC		WT
nhr-40-B	RS3721	tu1508	/	11bp del		AAAGGG-----------ATCTTCTCGC	WT
nhr-40-A/B	RS3723	tu1510 (A) & tu1511(B)	1bp del	1bp del	AAAGGGTGC-TTCGGGGAT	AAAGGGTGC-TTCGGGGATC	Te-like instead of Te
nhr-40-A/B; nag-A/B	RS3942	tu1662	1bp del	1bp del	AGGGTGC-TTCGGGG	AGGGTGC-TTCGGGG	St-like and Te-like
sult-1-A	RS3993	tu1682	11bp del	/	ACGTAGACG-----------TGGGCGCATG		WT
sult-1-B	RS3994	tu1683	/	17bp del		TGCACACG-----------------CGGGCGCAT	WT
sult-1-A/B		tu1682 (A) & tu1683 (B)	11bp del	17bp del	ACGTAGACG-----------TGGGCGCATG	TGCACACG-----------------CGGGCGCAT	Eu instead of St
sul-2-A/B; nag-A/B; nhr-40-A/B	RS4438	tu2052	8bp del	17bp del	ACCAAGT--------GCCTACTC	TAGGCA-----------------TTCGAG	All-St
