# Supplementary material for: Functional divergence of conserved developmental plasticity genes between two distantly related nematodes
Source: Sci Rep. 2025 Aug 5;15:28518. doi: 10.1038/s41598-025-14207-5 (PMC12325724; doi:10.1038/s41598-025-14207-5)
Supplement: Supplementary file 9 — Supplementary Information 9. [file 41598_2025_14207_MOESM9_ESM.pdf]

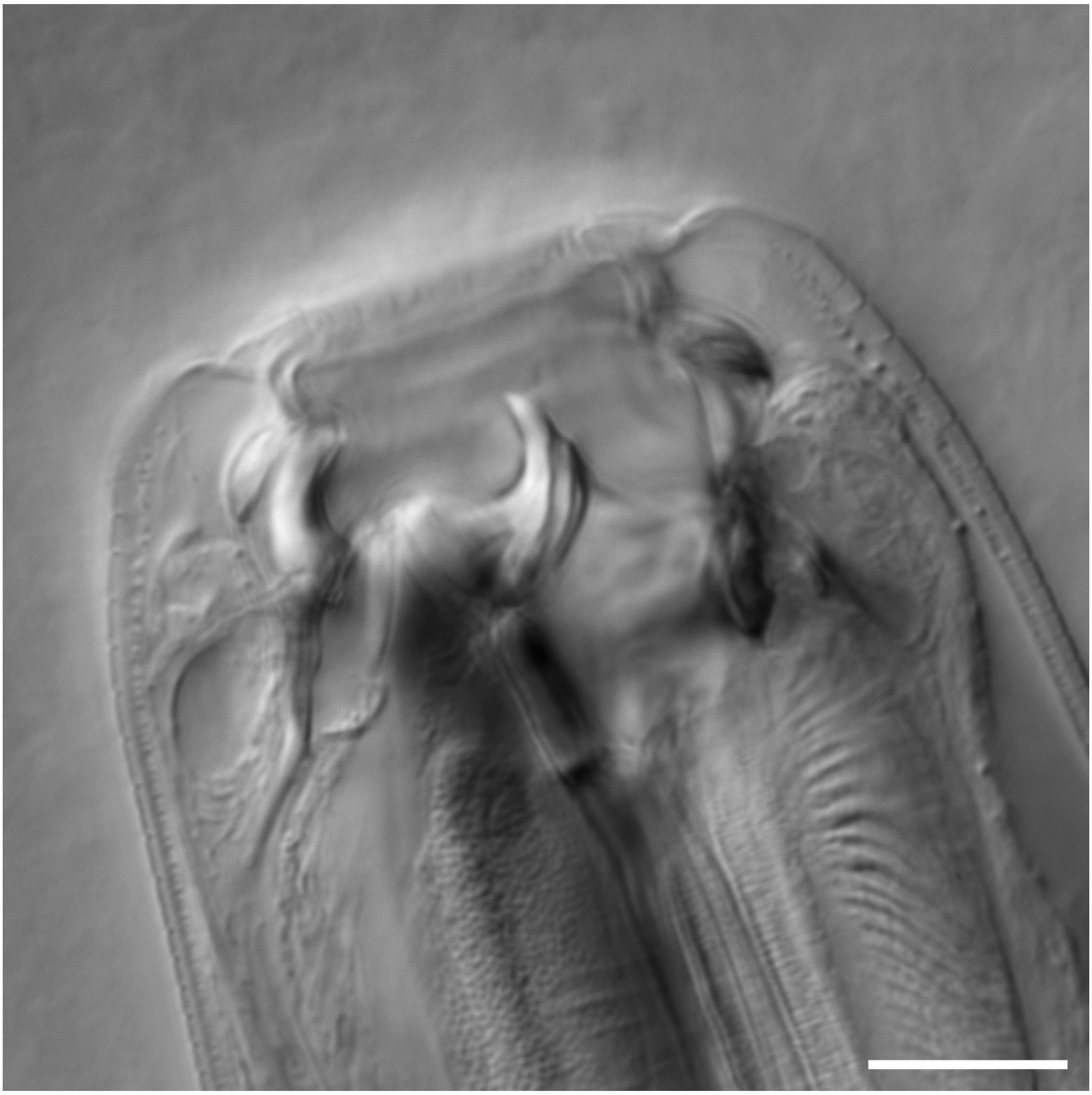

Figure S1: The *ssu-1-A/B* double mutant knockout can become Te on *P. camemberti* as seen in this DIC image. Scale bar: 10 µm.
